# A Three-rooted Mandibular Second Premolar: A Case Report

**DOI:** 10.5681/joddd.2014.034

**Published:** 2014-09-17

**Authors:** Zahra Fathi, Saeed Rahimi, Reza Tavakoli, Mahsa Amini

**Affiliations:** ^1^Post-graduate Student, Department of Endodontics, Faculty of Dentistry, Tabriz University of Medical Sciences, Tabriz, Iran; ^2^Professor, Department of Endodontics, Dental and Periodontal Research Center, Faculty of Dentistry, Tabriz University of Medical Sciences, Tabriz, Iran; ^3^Post-graduate Student, Department of Oral Medicine, Faculty of Dentistry, Tabriz University of Medical Sciences, Tabriz, Iran

**Keywords:** Dental pulp cavity abnormality, bicuspids, three-rooted premolars

## Abstract

Presence of extra roots and canals should be considered before initiation of root canal treatment for the success of endodontic treatment. A mandibular second premolar with three separate roots is very rare and its prevalence has been reported to be around 0.1%. This case report explains non-surgical endodontic treatment of a mandibular second premolar with three separate roots and three separate mesiobuccal, midbuccal, and lingual canal orifices. Close attention to anatomic variations, thorough radiographic examinations, thorough evaluation of the pulp chamber floor, and use of magnifying and optical devices have been recommended for the success of endodontic treatment of mandibular second premolars with complicated root canal system anatomy.

## Introduction

Thorough knowledge of the anatomy and morphology of the root canal system is necessary for successful endodontic treatment. A study by Slowey has shown that mandibular premolars are the most difficult teeth for endodontic treatment due to their complicated root canal anatomy.^1^

Various studies have shown the effect of gender and ethnicity on anatomic variations and complexity of the root canal system in mandibular second premolars.^2^ In this context, the prevalence of mandibular premolars with more than one canal has been reported to be significantly higher in blacks compared to whites.^3^ Serman and Hasselgren^4^ reported a higher prevalence of several roots and canals in mandibular premolars in males compared to females.

In a study by Cleghorn et al^2^almost all of the mandibular second premolars had one root (99.6%), 0.3% of them had two roots, and only 0.1% had three roots. Anyway, a thorough knowledge of the anatomy of root canal, a meticulous radiographic interpretation, and a proper access cavity are necessary to increase the success rate of endodontic treatment of these teeth.

The present case report explains therapeutic recommendations and the procedural steps of the endodontic treatment of a rare case of mandibular second premolar with three separate roots.

## Case report 

**Figure 1.**
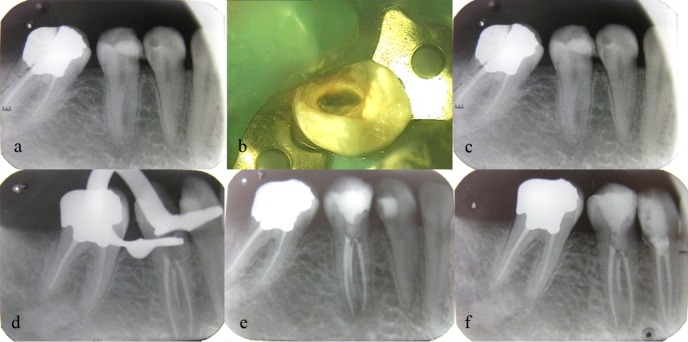
a) Preoperative radiograph of three-rooted second mandibular premolar. b) Evaluation of access cavity under a surgical microscope reveals two buccal orifices and one lingual orifice. c) Determination of working lengths by radiography. d) Immediate postoperative radiograph showing three separate roots. e) Three-month follow-up. f) Six-month follow-up.

The patient was a 30-year-old female with no history of any systemic diseases. The patient had referred to the Department of Endodontics, Tabriz Faculty of Dentistry, with a chief complaint of pain in the posterior area of the right lower arch. Clinical evaluations revealed a carious lesion in the mandibular second premolar. Pulp vitality tests showed sensitivity to heat, cold, and electric pulp tests. Sensitivity to percussion was in the normal range. Radiographic evaluation revealed normal periodontium and presence of more than one root (Figure 1a).

The pulp was diagnosed with irreversible pulpitis with normal periradicular tissues. A two-session endodontic treatment was planned. Isolation was achieved by rubber dam after local anesthesia with 2% lidocaine and 1:100,000 epinephrine. To gain sufficient access to the canals, the conventional access opening was modified in the way that it was wider mesially.

Evaluation under a surgical microscope (OPMI Pice Dental Microscope, Zeiss, Oberkochen, Germany) at a magnification of ×10 revealed three separate mesiobuccal, midbuccal and lingual orifices (Figure 1b).

Working length was determined with an apex locator (Root ZX, J Morita Inc, USA) and confirmed by radiography (Figure 1c). Debridement and shaping of the canals were carried out by RaCe rotary files (FKG Dentaire, La-Chaux de Fonds, Switzerland) using the crown-down technique up to the final sizes of 0.04/30, 0.04/30 and 0.06/25 in the lingual, mesiobuccal and midbuccal canals, respectively. The canals were irrigated with 2.5% sodium hypochlorite and 17% EDTA during instrumentation.

After final irrigation with normal saline solution, the canals were dried with paper points and obturated with gutta-percha and AH26 (Dentsply, De Trey, Konstanz, Germany) sealer using lateral compaction technique (Figure 1d). The tooth was asymptomatic with normal radicular conditions at 3- and 6-month follow-ups (Figure 1e, f).

## Discussion

Mandibular second premolar is one of the most difficult teeth for endodontic treatment due to wide variations in the morphology of its root canal system.^1^ Meticulous radiographic evaluation using straight tube angulation and changes in horizontal angulation will help exactly diagnose the number of roots and canals in premolar teeth. A “fast break” of the canal on parallel radiographs might be an indication of the presence of more than one canal.^5^ Martinez-Lozano et al^6^ reported that a change of 40° in the horizontal x-ray tube angulation can contribute to the identification of an extra canal in mandibular second premolars.

In the present case, before the initiation of the treatment, the tooth was determined to have two mesial and distal roots on the radiograph with direct tube angulation; however, a change in the horizontal angulation of the x-ray tube revealed three separate roots (Figure 1a).

Use of magnification with the help of a loupe or a microscope and visual enhancement with the use of fiber optics, use of sodium hypochlorite bubble technique and staining might help locate additional canals.^7^

In this case, a surgical microscope was used to enhance visualization, and the anatomic map of the pulp chamber floor was used to locate canal orifices (Figure 1b).

Previous studies have shown the presence of one orifice on the lingual aspect and two orifices on the buccal aspect.^8^ In the present case, one canal orifice was on the lingual aspect, one canal orifice was on the buccal aspect, and the third orifice was located mesial to the two other orifices (Figure 1b).

After working lengths were determined using an apex locator, they were confirmed by radiography to achieve more reliable treatment results. All the three canals were obturated using lateral condensation technique.

The advantages of cone-beam computed tomography (CBCT) as a diagnostic tool for effective assessment of root canal anatomy and morphology has been confirmed.^9^ In this case by using the patient CBCT (Planmeca OY, Helsinki, Finland) and a dental software program (Planmeca Romexis Viewer) that had been prepared for implant insertion in posterior edentulous sites, presence of three obturated root canals with three separate foramina in the axial section and cephalometric image was confirmed (Figure 1a,b). Concerning bilateral anatomic variations, Sabala et al^10^ reported that abnormal morphology of the roots is bilateral in almost 90% of the cases and the rarer the prevalence the higher the odds of bilateral occurrence. In the present case, due to the extraction of the second premolar on the left side it was not possible to evaluate the morphology of the second premolar on the left side. However, the root canal morphology of mandibular first premolar on the left side was similar to that on the right side.

Although it is very rare for a mandibular second premolar to have three roots, each case should be carefully examined radiographically and clinically to locate all the root canals.
